# A curriculum map of colorectal surgery: an assessment of training expectations and the reality

**DOI:** 10.1186/1753-6561-6-S4-O4

**Published:** 2012-07-09

**Authors:** C May

**Affiliations:** 1 University of Manchester, UK

## Introduction

There are national guidelines for the curriculum of each surgical specialty, encompassing set competencies and attitudes expected of trainees at each level. Conventionally assessment is based on the trainee and not delivery of the curriculum. By performing the curriculum map it reviews the trainees' ability and also the quality and breadth of delivery by seniors.

## Methods

The initial phase involved comparing trainees expected skill level against their subjective competency in a number of areas. Data was then collected, covering a twelve month period, tallying the number of elective procedures carried out. By comparing this data against expected trainee progression it was possible to assess if there was adequate exposure for such a level to be achieved.

## Results

The subjective data collected from the trainees themselves suggested they were comfortably achieving the set competencies within their training band. Despite collecting data across four consultants elective theatre lists there were a number of procedures, detailed within the set curriculum, that had not been performed during the twelve month period. Alongside this there were a number of procedures that were performed in such low numbers that trainees would not receive adequate exposure and training opportunities.

## Conclusions

Conducting the curriculum map highlighted key areas where trainees might be missing out on valuable learning opportunities which potentially may not have been picked up on until formal assessment. The methods used for conducting a curriculum map are not without flaws though. Using data that requires subjective assessment of skills can lead to false beliefs about efficacy of curriculum delivery. Ultimately it is therefore essential that formal trainee assessments are running concurrently with curriculum maps at each site.

